# Supplementary material for: Space-time superoscillations
Source: Nat Commun. 2026 Feb 2;17:2053. doi: 10.1038/s41467-025-68260-9 (PMC12946195; doi:10.1038/s41467-025-68260-9)
Supplement: Supplementary file 1 — Supplementary Information [file 41467_2025_68260_MOESM1_ESM.pdf]

Supplementary Materials for

Space-Time Superoscillations

Yijie Shen[1,2], Nikitas Papasimakis[3], Nikolay I. Zheludev[3,4]

1. Centre for Disruptive Photonic Technologies, School of Physical and Mathematical Sciences, Nanyang Technological University, Singapore 637371, Singapore
2. School of Electrical and Electronic Engineering, Nanyang Technological University, Singapore 639798, Singapore
3. Optoelectronics Research Centre & Centre for Photonic Metamaterials, University of Southampton, Southampton SO17 1BJ, UK
4. Institute for Advanced Study, Texas A&M University, USA

Supplementary text

S1. Space-time solution of supertoroidal pulses

To obtain the exact space-time nonseparable solution of electromagnetic waves, we start from the scalar generating function derived from modified power spectrum method [1,2]:

$$f = f_0 \frac{e^{-s/q_3}}{(q_1 + i\tau)(s + q_2)^\alpha} \tag{S1}$$

Where $s \equiv r^2/(q_1 + i\tau) - i\sigma$, $\tau = z - ct$, $\sigma = z + ct$, $r = \sqrt{x^2 + y^2}$, (x, y, z) is the spatial coordinate, t is time, $c = 1/\sqrt{\mu_0 \varepsilon_0}$ is the speed of light, the parameters q_1, q_2, q_3 are real positive with units of length, and the real dimensionless parameter α must satisfy $\alpha \geq 1$ in order for the electromagnetic pulse to fulfill finite energy. In conventional method, it always assumes that $q_3 \to \infty$ and $\alpha = 1$. Here we break the limit of the parameter α, that can be any real number no less than one in our new derivation. The scalar generating function is given by:

$$\begin{aligned} f &= f_0 \frac{1}{(q_1 + i\tau)\left(\dfrac{r^2}{q_1 + i\tau} - i\sigma + q_2\right)^\alpha} \\ &= f_0 \frac{(q_1 + i\tau)^{\alpha-1}}{\left[r^2 + (q_1 + i\tau)(q_2 - i\sigma)\right]^\alpha} \end{aligned} \tag{S2}$$

The doughnut-like pulse is derived under a curled vector Hertz potential $\mathbf{\Pi} = \nabla \times \hat{\mathbf{z}} f(\mathbf{r}, t)$ in cylindrical coordinate (r, θ, z), the TE-mode electromagnetic field can be generated from Hertz potential by

$$\begin{cases} \mathbf{E}(\mathbf{r}, t) = -\mu_0 \dfrac{\partial}{\partial t} \nabla \times \mathbf{\Pi} = \hat{\boldsymbol{\theta}} \mu_0 \partial_r \partial_t f \\ \mathbf{H}(\mathbf{r}, t) = \nabla \times (\nabla \times \mathbf{\Pi}) = \hat{\mathbf{r}} \partial_r \partial_z f + \hat{\mathbf{z}} \left(\partial_z^2 - \dfrac{1}{c^2} \partial_t^2 \right) f \end{cases} \tag{S3}$$

For this pulse solution, the electric field is purely azimuthally polarized, and the magnetic field is along the radial and longitudinal directions with no azimuthal component. The azimuthally polarized electric field of supertoroidal pulses is derived as [3]:

$$E=\mu_0\partial_z\partial_t f=\mu_0 f_0\partial_\rho\partial_t \frac{(q_1+i\tau)^{\alpha-1}}{\left[r^2+(q_1+i\tau)(q_2-i\sigma)\right]^\alpha}$$

$$=\mu_0 f_0\left\{\frac{-2\alpha(\alpha+1)icr(q_1+i\tau)^{\alpha-1}(q_1+q_2-2ict)}{\left[r^2+(q_1+i\tau)(q_2-i\sigma)\right]^{\alpha+2}}+\frac{2(\alpha-1)\alpha icr(q_1+i\tau)^{\alpha-2}}{\left[r^2+(q_1+i\tau)(q_2-i\sigma)\right]^{\alpha+1}}\right\} \quad \text{(S4)}$$

$$=f_0\sqrt{\frac{\mu_0}{\varepsilon_0}}\left\{\frac{-2\alpha(\alpha+1)ir(q_1+i\tau)^{\alpha-1}(q_1+q_2-2ict)}{\left[r^2+(q_1+i\tau)(q_2-i\sigma)\right]^{\alpha+2}}+\frac{2(\alpha-1)\alpha ir(q_1+i\tau)^{\alpha-2}}{\left[r^2+(q_1+i\tau)(q_2-i\sigma)\right]^{\alpha+1}}\right\}$$

This equation (S4) is the expression of high-order flying doughnut pulse. Note that when $\alpha=1$, the equation (S4) will be reduced into the expression of fundamental flying doughnut pulse [2]. For the paraxial limit condition, $q_2 \gg q_1$, and neglecting extremely small value of the second term, the equation (S4) can be simplified as:

$$E=-2\alpha(\alpha+1)if_0\sqrt{\frac{\mu_0}{\varepsilon_0}}\frac{r(q_1+i\tau)^{\alpha-1}(q_2-2ict)}{\left[r^2+(q_1+i\tau)(q_2-i\sigma)\right]^{\alpha+2}} \quad \text{(S5)}$$

Considering the field is a very short propagating pulse at the speed of *c*, and $\tau=z-ct$ represents the local time, we can use the approximation of $z\doteq ct$ to evaluate the $\sigma=z+ct\doteq 2z$, then the electric field can be derived as:

$$E=-2\alpha(\alpha+1)if_0\sqrt{\frac{\mu_0}{\varepsilon_0}}\frac{r(q_1+i\tau)^{\alpha-1}(q_2-i2z)}{\left[r^2+(q_1+i\tau)(q_2-i2z)\right]^{\alpha+2}}$$

$$=-2\alpha(\alpha+1)if_0\sqrt{\frac{\mu_0}{\varepsilon_0}}r(q_1+i\tau)^{\alpha-1}\frac{1}{(q_2-i2z)^{\alpha+1}}\frac{1}{\left[\frac{r^2}{q_2-i2z}+(q_1+i\tau)\right]^{\alpha+2}}$$

$$=-2\alpha(\alpha+1)if_0\sqrt{\frac{\mu_0}{\varepsilon_0}}r(q_1+i\tau)^{\alpha-1}\left(\frac{q_2+i2z}{4z^2+q_2^2}\right)^{\alpha+1}\frac{1}{\left[q_1+\frac{r^2(q_2+i2z)}{4z^2+q_2^2}+i\tau\right]^{\alpha+2}} \quad \text{(S6)}$$

$$=-2\alpha(\alpha+1)if_0\sqrt{\frac{\mu_0}{\varepsilon_0}}r(q_1+i\tau)^{\alpha-1}\left(\frac{q_2+i2z}{4z^2+q_2^2}\right)^{\alpha+1}\frac{1}{\left[q_1+\frac{q_2r^2}{4z^2+q_2^2}+i\left(\tau+\frac{2zr^2}{4z^2+q_2^2}\right)\right]^{\alpha+2}}$$

Here we define the notations of radius of curvature, $R(z)$, and beam waist profile, $w(z)$, as

$$R(z)=z\left[1+\left(\frac{z_0}{z}\right)^2\right]=\frac{4z^2+q_2^2}{4z} \quad \text{(S7)}$$

$$w^2(z)=w_0^2\left[1+\left(\frac{z}{z_0}\right)^2\right]=\frac{q_1}{2q_2}\left(4z^2+q_2^2\right) \quad \text{(S8)}$$

with the Rayleigh length and basic waist constant given by $z_0 = \frac{q_2}{2}$ and $w_0^2 = \frac{q_1 q_2}{2} = q_1 z_0$.
Substitute these two notations and equations (S7) and (S8) into equation (S6), we can simplify electric field expression as:

$$
\begin{aligned}
E &= -2\alpha(\alpha+1) i f_0 \sqrt{\frac{\mu_0}{\varepsilon_0}} r (q_1 + i\tau)^{\alpha-1} \left[\frac{q_2 \left(1 + i\frac{2z}{q_2}\right)}{\frac{2q_2}{q_1} w^2} \right]^{\alpha+1} \frac{1}{\left[q_1 \left(1 + \frac{r^2}{2w^2}\right) + i\left(\tau + \frac{r^2}{2R}\right) \right]^{\alpha+2}} \\
&= -2\alpha(\alpha+1) i f_0 \sqrt{\frac{\mu_0}{\varepsilon_0}} r (q_1 + i\tau)^{\alpha-1} \left[\frac{w_0^2 \left(1 + i\frac{z}{z_0}\right)}{2 z_0 w^2} \right]^{\alpha+1} \frac{1}{\left[q_1 \left(1 + \frac{r^2}{2w^2}\right) + i\left(\tau + \frac{r^2}{2R}\right) \right]^{\alpha+2}} \qquad \text{(S9)} \\
&= -\frac{\alpha(\alpha+1)}{2^\alpha} i f_0 \sqrt{\frac{\mu_0}{\varepsilon_0}} r (q_1 + i\tau)^{\alpha-1} \frac{w_0^{2(\alpha+1)}}{z_0^{\alpha+1} w^{2(\alpha+1)}} \left(1 + i\frac{z}{z_0}\right)^{\alpha+1} \frac{1}{\left[q_1 \left(1 + \frac{r^2}{2w^2}\right) + i\left(\tau + \frac{r^2}{2R}\right) \right]^{\alpha+2}}
\end{aligned}
$$

Applying Taylor expansion of $\sqrt{1+x^2}\exp\left(i\tan^{-1}x\right) = 1 + ix + O\left(\frac{x^2}{\sqrt{2}}\right)$, i.e. the approximation of $1 + ix \doteq \sqrt{1+x^2}\exp\left(i\tan^{-1}x\right)$, the numerator term in equation (S9) can be rewritten as:

$$
\left(1 + i\frac{z}{z_0}\right)^{\alpha+1} = \left\{ \sqrt{1 + \left(\frac{z}{z_0}\right)^2} \exp\left[i \cdot \tan^{-1}\left(\frac{z}{z_0}\right) \right] \right\}^{\alpha+1} = \frac{w^{\alpha+1}}{w_0^{\alpha+1}} \exp\left[i(\alpha+1)\phi(z) \right] \qquad \text{(S10)}
$$

Where the $\phi(z) = \tan^{-1}\left(\frac{z}{z_0}\right)$ is the Gouy phase. Substitute equation (S10) into (S9) to carry on the simplification:

$$
\begin{aligned}
E &= -\frac{\alpha(\alpha+1)}{2^\alpha} i f_0 \sqrt{\frac{\mu_0}{\varepsilon_0}} r (q_1 + i\tau)^{\alpha-1} \frac{w_0^{2(\alpha+1)}}{z_0^{\alpha+1} w^{2(\alpha+1)}} \exp\left[i(\alpha+1)\phi(z) \right] \cdot \frac{1}{\left[q_1 \left(1 + \frac{r^2}{2w^2}\right) + i\left(\tau + \frac{r^2}{2R}\right) \right]^{\alpha+2}} \\
&= -\frac{\alpha(\alpha+1)}{2^\alpha} i f_0 \sqrt{\frac{\mu_0}{\varepsilon_0}} \frac{w_0^{\alpha+1} r (q_1 + i\tau)^{\alpha-1}}{z_0^{\alpha+1} w^{\alpha+1}} \cdot \frac{\left[q_1 \left(1 + \frac{r^2}{2w^2}\right) - i\left(\tau + \frac{r^2}{2R}\right) \right]^{\alpha+2}}{\left[\left(q_1 \left(1 + \frac{r^2}{2w^2}\right) \right)^2 + \left(\tau + \frac{r^2}{2R}\right)^2 \right]^{\alpha+2}} \cdot \exp\left[i(\alpha+1)\phi(z) \right]
\end{aligned}
$$

(S11)

Define the notations of radially scaled local time, $T(\mathbf{r},\tau)$, as

$$T=\frac{-\left(\tau+\frac{r^2}{2R}\right)}{q_1\left(1+\frac{r^2}{2w^2}\right)}=\frac{c\left(t-\frac{z+r^2/2R}{c}\right)}{q_1\left(1+\frac{r^2}{2w^2}\right)} \tag{S12}$$

Substitute equation (S12) into (S11) to carry on the simplification:

$$\begin{aligned}E&=-\alpha(\alpha+1)if_0\sqrt{\frac{\mu_0}{\varepsilon_0}}\frac{r(q_1+i\tau)^{\alpha-1}}{2^\alpha\left(z^2+z_0^2\right)^{(\alpha+1)/2}}\cdot\frac{\left[q_1\left(1+\frac{r^2}{2w^2}\right)\right]^{\alpha+2}(1+iT)^{\alpha+2}}{\left[\left(q_1\left(1+\frac{r^2}{2w^2}\right)\right)^2\left(1+T^2\right)\right]^{\alpha+2}}\cdot\exp\left[i(\alpha+1)\phi(z)\right]\\&=-\frac{\alpha(\alpha+1)}{2^\alpha}if_0\sqrt{\frac{\mu_0}{\varepsilon_0}}\frac{w_0^{\alpha+1}r(q_1+i\tau)^{\alpha-1}}{z_0^{\alpha+1}w^{\alpha+1}}\cdot\frac{(1+iT)^{\alpha+2}}{\left(q_1\left(1+\frac{r^2}{2w^2}\right)\right)^{\alpha+2}\left(1+T^2\right)^{\alpha+2}}\cdot\exp\left[i(\alpha+1)\phi(z)\right]\end{aligned} \tag{S13}$$

Applying the Taylor approximation of equation to simplify the numerator term in equation (S13), we get:

$$(1+iT)^{\alpha+2}\doteq\left[\sqrt{1+T^2}\exp\left(i\tan^{-1}T\right)\right]^{\alpha+2}=\left(1+T^2\right)^{(\alpha+2)/2}\exp\left[i\cdot(\alpha+2)\tan^{-1}T\right] \tag{S14}$$

To simplify the numerator term in equation (S13), we carry on the simplification of the electric field expression:

$$\begin{aligned}E&=-\frac{\alpha(\alpha+1)}{2^\alpha}if_0\sqrt{\frac{\mu_0}{\varepsilon_0}}\frac{w_0^{\alpha+1}r(q_1+i\tau)^{\alpha-1}}{z_0^{\alpha+1}w^{\alpha+1}}\cdot\frac{\left(1+T^2\right)^{(\alpha+2)/2}\exp\left[i\cdot(\alpha+2)\tan^{-1}T\right]}{\left(q_1\left(1+\frac{r^2}{2w^2}\right)\right)^{\alpha+2}\left(1+T^2\right)^{\alpha+2}}\cdot\exp\left[i(\alpha+1)\phi(z)\right]\\&=-\frac{\alpha(\alpha+1)}{2^\alpha}if_0\sqrt{\frac{\mu_0}{\varepsilon_0}}\frac{w_0^{\alpha+1}r(q_1+i\tau)^{\alpha-1}}{z_0^{\alpha+1}w^{\alpha+1}\left(q_1\left(1+\frac{r^2}{2w^2}\right)\right)^{\alpha+2}\left(1+T^2\right)^{(\alpha+2)/2}}\cdot\exp\left\{i\left[(\alpha+2)\tan^{-1}T+(\alpha+1)\phi(z)\right]\right\}\end{aligned} \tag{S15}$$

The numerator term in equation (S15) can be further simplified by using the Taylor approximation to separate the amplitude and phase terms as:

$$\begin{aligned}(q_1+i\tau)^{\alpha-1}&=\left[q_1\left(1+i\frac{\tau}{q_1}\right)\right]^{\alpha-1}=\left(q_1\sqrt{1+\left(\frac{\tau}{q_1}\right)^2}\exp\left[i\tan^{-1}\left(\frac{\tau}{q_1}\right)\right]\right)^{\alpha-1}\\&=\left(q_1^2+\tau^2\right)^{(\alpha-1)/2}\exp\left[i(\alpha-1)\tan^{-1}\left(\frac{\tau}{q_1}\right)\right]\end{aligned} \tag{S16}$$

Substitute equation (S16) into (S15) to carry on the derivation:

$$E = -\frac{\alpha(\alpha+1)}{2^{\alpha}} i f_0 \sqrt{\frac{\mu_0}{\varepsilon_0}} \frac{w_0^{\alpha+1} r \left(q_1^2+\tau^2\right)^{(\alpha-1)/2}}{z_0^{\alpha+1} w^{\alpha+1} \left(q_1\left(1+\frac{r^2}{2w^2}\right)\right)^{\alpha+2} \left(1+T^2\right)^{(\alpha+2)/2}} \cdot \times \exp\left\{ i\left[(\alpha-1)\tan^{-1}\left(\frac{\tau}{q_1}\right) + (\alpha+2)\tan^{-1} T + (\alpha+1)\phi(z) \right]\right\} \tag{S17}$$

Define the notations of generalized local-time amplitude, $A_\alpha(\mathbf{r},\tau)$, and generalized local-time wavenumber, $k_\alpha(\mathbf{r},\tau)$, as

$$A_\alpha(\mathbf{r},\tau) = -f_0\sqrt{\frac{\mu_0}{\varepsilon_0}} \frac{\left(q_1^2+\tau^2\right)^{(\alpha-1)/2}}{q_1^{\alpha+2}(T^2+1)^{(\alpha+2)/2}} = \frac{-f_0\mu_0 c\left(q_1^2+\tau^2\right)^{(\alpha-1)/2}}{q_1^{\alpha+2}(T^2+1)^{(\alpha+2)/2}} \tag{S18}$$

$$k_\alpha(\mathbf{r},\tau) = (\alpha-1)\tan^{-1}\left(\frac{\tau}{q_1}\right) + (\alpha+2)\tan^{-1} T \tag{S19}$$

Substitute equations (S18) and (S19) into (S17), the final closed-form amplitude-phase expression of supertoroidal pulses is given by:

$$E = i\frac{\alpha(\alpha+1) w_0^{\alpha+1} r A_\alpha(\mathbf{r},\tau)}{2^{\alpha} z_0^{\alpha+1} w^{\alpha+1}\left(1+\frac{r^2}{2w^2}\right)^{\alpha+2}} \exp\left\{ i[k_\alpha(\mathbf{r},\tau) + (\alpha+1)\phi(z)]\right\} \tag{S20}$$

Note that, the amplitude spatiotemporal envelop is only related to τ ($z-ct$) for spatiotemporal evolution which means the theoretical group velocity of the pulse center is a constant of c, independent with α. The distribution of amplitude $|E|$ and phase Arg(E) of supertoroidal pulse can be calculated from this equation. With the similar derivation, the expressions for the magnetic field, including radial and longitudinal components, can be given as:

$$\begin{aligned} H_r &= \partial_r\partial_z f = f_0 \partial_r\partial_z \frac{\left(q_1+i\tau\right)^{\alpha-1}}{\left[r^2+\left(q_1+i\tau\right)\left(q_2-i\sigma\right)\right]^{\alpha}} \\ &= f_0\left\{ \frac{2\alpha(\alpha+1) i r (q_1+i\tau)^{\alpha-1}(q_2-q_1-2iz)}{\left[r^2+\left(q_1+i\tau\right)\left(q_2-i\sigma\right)\right]^{\alpha+2}} - \frac{2(\alpha-1)\alpha i r (q_1+i\tau)^{\alpha-2}}{\left[r^2+\left(q_1+i\tau\right)\left(q_2-i\sigma\right)\right]^{\alpha+1}} \right\} \end{aligned} \tag{S21}$$

$$\begin{aligned} H_z &= \left(\partial_z^2 - \frac{1}{c^2}\partial_t^2\right) f = f_0\left(\partial_z^2 - \frac{1}{c^2}\partial_t^2\right) \frac{\left(q_1+i\tau\right)^{\alpha-1}}{\left[r^2+\left(q_1+i\tau\right)\left(q_2-i\sigma\right)\right]^{\alpha}} \\ &= f_0\left\{ \frac{-4\alpha(q_1+i\tau)^{\alpha-1}\left[r^2-\alpha\left(q_1+i\tau\right)\left(q_2-i\sigma\right)\right]}{\left[r^2+\left(q_1+i\tau\right)\left(q_2-i\sigma\right)\right]^{\alpha+2}} - \frac{4(\alpha-1)\alpha(q_1+i\tau)^{\alpha-2}\left(q_2-i\sigma\right)}{\left[r^2+\left(q_1+i\tau\right)\left(q_2-i\sigma\right)\right]^{\alpha+1}} \right\} \end{aligned} \tag{S22}$$

and the radial magnetic component can be derived into the amplitude-phase formation

$$H_r = -i\sqrt{\frac{\varepsilon_0}{\mu_0}} \frac{\alpha(\alpha+1)w_0^{\alpha+1} r A_\alpha(\mathbf{r},\tau)}{2^\alpha z_0^{\alpha+1} w^{\alpha+1}\left(1+\frac{r^2}{2w^2}\right)^{\alpha+2}} \exp\{i[k_\alpha(\mathbf{r},\tau)+(\alpha+1)\phi(z)]\} \tag{S23}$$

Based on the transverse components of the electromagnetic field, Eqs. (S20) and (S23), The transverse electromagnetic field can reach a more compact formation if we create a notation of the complex amplitude of $A_\alpha = i\frac{\alpha(\alpha+1)w_0^{\alpha+1} r A_\alpha(\mathbf{r},\tau)}{2^\alpha z_0^{\alpha+1} w^{\alpha+1}\left(1+r^2/(2w^2)\right)^{\alpha+2}}$, the unified expression of the transverse field can be simplified as:

$$\mathbf{\Psi}_\perp = \begin{bmatrix}\mathbf{E}_\perp \\ \mathbf{H}_\perp\end{bmatrix} = \begin{bmatrix}\hat{\boldsymbol{\theta}} \\ \sqrt{\frac{\varepsilon_0}{\mu_0}}\hat{\mathbf{r}}\end{bmatrix} A_\alpha \exp\{i[k_\alpha(\mathbf{r},\tau)+(\alpha+1)\phi(z)]\} \tag{S24}$$

Figure S1 shows isosurfaces and log modulus of the spatiotemporal electric field profiles of examples STP cases.

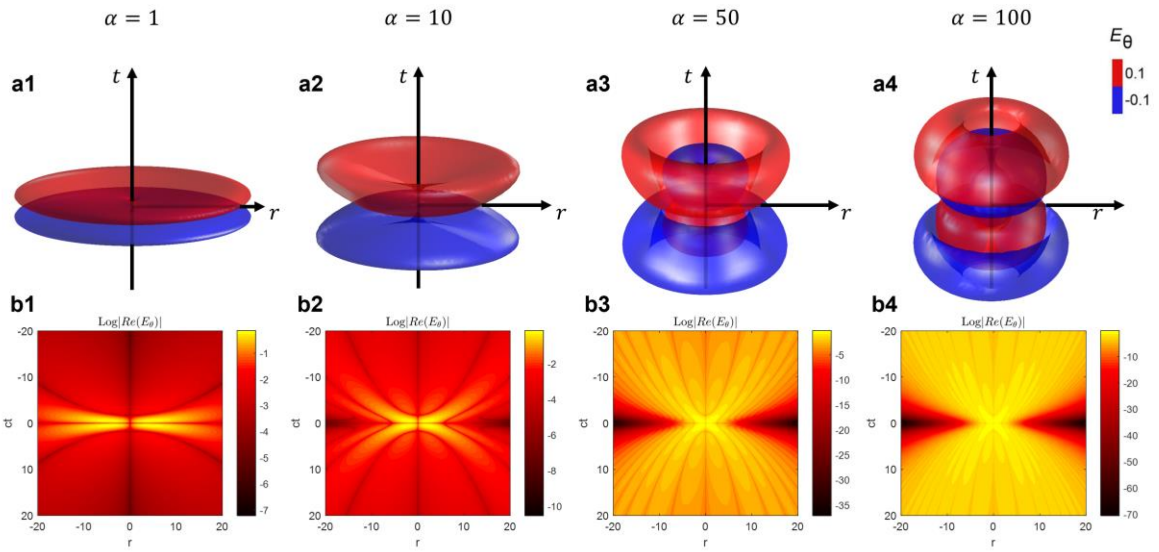

Figure S1: (a1-a4) Isosurfaces of the spatiotemporal electric field, $E_\theta(r,z{=}0,t)$ of STPs in the focal plane for α =1,10,50,100, respectively, and (b1-b4) the corresponding log modulus ($|E_\theta(r,z{=}0,t)|$) distributions.

S2. Space-Time Superoscillations in band-limited STPs

The spectrum of a supertoroidal light pulse (STP), given by $E(r, z, t)$, can be directly obtained by Fourier transform from space-time (r, z, t) to spatiotemporal frequency (f_r, f_z, f) domain.

$$\tilde{E}(f_r, f_z, f) = \int_{-\infty}^{\infty}\int_{-\infty}^{\infty}\int_{-\infty}^{\infty} E(r,z,t)\exp\left[-i2\pi(f_r r + f_z z + ft)\right] J_0\left(2\pi f_r r\right) r dr dz dt \tag{S25}$$

Limited by the solution of Maxwell's equations, the entire spectrum of a space-time light pulse must be distributed on the surface of light cone, i.e the conic surface with unit slope of its

generatrix in the coordinate (k_r, k_z, ω/c), where the spatial wavevectors are related to the spatial frequencies by $k_r=2\pi f_r/c$ and $k_r=2\pi f_z/c$, and temporal frequency is related to the angular frequency by $\omega=2\pi f$. In numerical calculation, we should make sure the range of space-time is set large enough to cover effective energy distribution (the energy at the boundary is 1/1000 less than the entire energy). Figure S1 shows the theoretical results of the spectra of several supertoroidal pulses with various of orders. Although the entire spectrum of a supertoroidal pulse must be located on the light cone, but the local spectrum of which does not necessary to fulfill this requirement and can be distributed over the limit of light cone, for example, the radial-temporal spectrum of a supertoroidal pulse at a specific propagation distance:

$$\tilde{E}(f_r, f) = \int_{-\infty}^{\infty}\int_{-\infty}^{\infty} E(r,0,t)\exp\left[-i2\pi(f_r r + ft)\right] J_0\left(2\pi f_r r\right) r dr dt \tag{S26}$$

as the results of Figure 3 in the main text show.

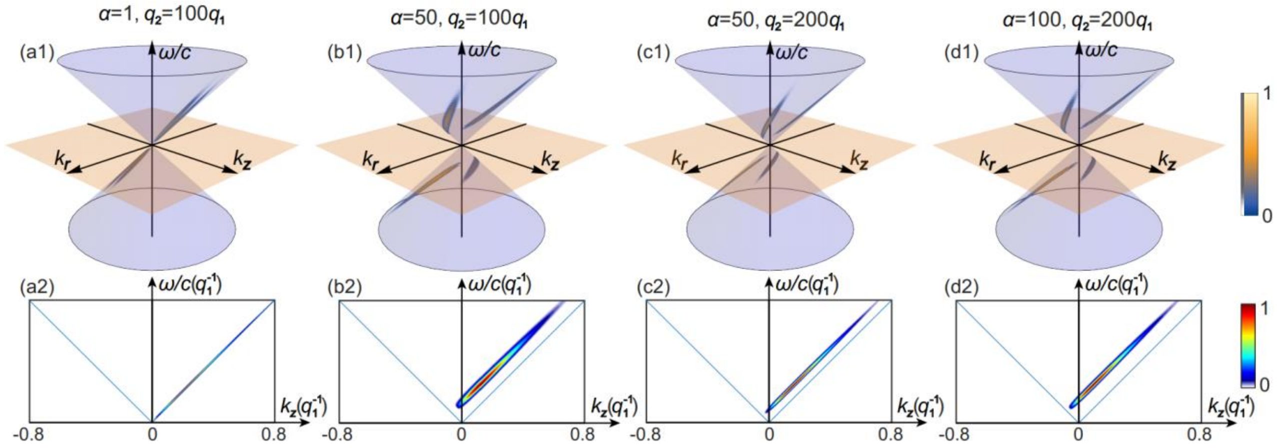

Figure S2: (a1-d1) The power spectra of various supertoroidal pulses in (k_r, k_z, ω/c) domain, and (a2-d2) their projection patterns onto the k_z–ω/c plane. The blue surface is the light cone. Parameters: (a) α=1, q_2=100q_1; (b) α=50, q_2=100q_1; (c) α=50, q_2=200q_1; (d) α=100, q_2=200q_1.

Theoretically, an ideal supertoroidal pulse is not exactly band-limited (boundary by zero-value), while the effective energy is localized in a finite region (exponentially decayed toward infinity). Therefore, we can still find the effective band-limited effect in such pulses. In the numerical calculation of results of Figure S2, the band limits for the k_r, k_z, ω/c are all set as 2.5, which will not impact on the reconstruction of spatiotemporal signal (inverse Fourier Transform):

$$\tilde{\tilde{E}}(r,z,t) = \int_{-\infty}^{\infty}\int_{-\infty}^{\infty}\int_{-\infty}^{\infty} \tilde{E}(f_r, f_z, f)\exp\left[i2\pi(f_r r + f_z z + ft)\right] J_0\left(2\pi f_r r\right) f_r df_r df_z df \tag{S27}$$

We compared the normalized reconstructed signal and theoretical signal and make sure the maximal amplitude difference between them is less than 0.01. In this case, the band-limited effect cannot be revealed in numerical calculation. In order to reveal the band-limited effect and study superoscillation effect, we proposed an approach of on-demand band-limited modulation onto a supertoroidal pulse, which is illustrated in Figure S3. We start from an ideal space-time signal of supertoroidal pulse (Figure S3(a)) and the entire spectrum can be numerical simulated by Fourier transformation (Figure S3(b)). In order to obtain a band-limited signal, we use the product of the

entire spectrum and a frequency dependent bump function to be as the spectrum of the modulated signal. The bump function is a continue function between 0 and 1, which can be defined as

$$B(f)=\begin{cases}1, & |f|<f_m-\delta \\ \exp\left[-\dfrac{1}{1-(f\mp f_m)^2/\delta^2}\right], & f_m-\delta<\pm f<f_m \\ 0, & \text{otherwise}\end{cases} \tag{S28}$$

Such a bump function is plotted in Figure S3(c), where f_m is the cutting-off frequency to set exact band limit and δ is a small frequency gap to ensure a physical smooth change of function (avoid non-physical step). Then the band-limited spectrum of the modulated signal can be expressed as:

$$\tilde{E}_c(f_r, f_z, f)=\tilde{E}(f_r, f_z, f)B(f) \tag{S29}$$

Figure S3(d) shows a simulated result of the band-limited spectrum with cutting-off frequency of f_m = 0.6 (c/q_1) and plotted onto the light cone. In our simulation, the δ is set as f_m/20. The corresponding band-limited spatiotemporal supertoroidal-like pulse can be obtained by inverse Fourier transform:

$$\tilde{\tilde{E}}_c(r,z,t)=\int_{-\infty}^{\infty}\int_{-\infty}^{\infty}\int_{-\infty}^{\infty}\tilde{E}_c(f_r, f_z, f)\exp\left[i2\pi(f_r r+f_z z+ft)\right]J_0\left(2\pi f_r r\right) f_r df_r df_z df \tag{S30}$$

Such a synthetic spatiotemporal pulse, we call band-limited supertoroidal-like pulse (arbitrary value of supertoroidal order α can be set in which), has exact band limit of spatial and temporal frequencies (f_r, f_z, f) with value of f_m, that can be arbitrarily tuned.

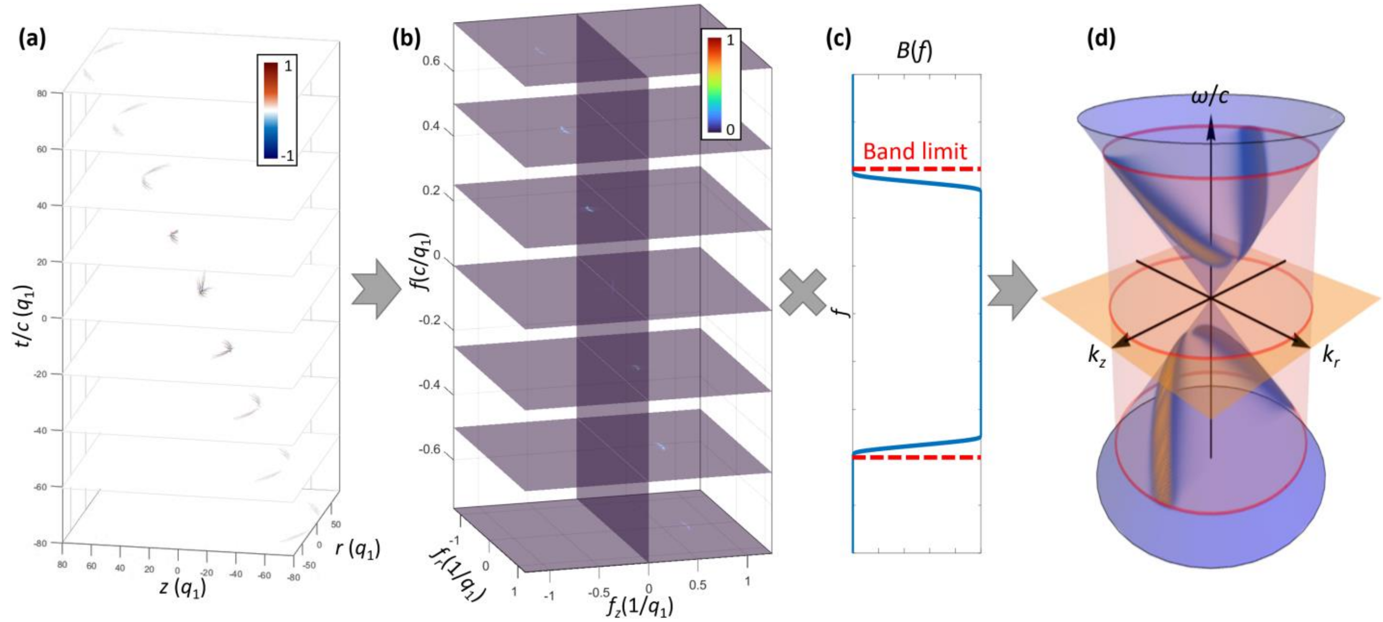

Figure S3: The process of setting band-limited modulation onto a supertoroidal pulse. (a) The space-time distributions of the amplitude of a supertoroidal pulse (α=20, q_2=50q_1), and (b) its numerically calculated entire spectrum. (c) The frequency dependent bump function with on-

demand band-limit set. (d) The band-limited entire spectrum is obtained by multiply the original entire spectrum and the bump function, which is plotted on a light cone with red line highlighting the band limit.

In order to qualitatively characterize the superoscillation of band-limited supertoroidal-like pulse, we numerically compared the oscillatory structures of the fastest locally oscillatory signal and the fastest harmonic Fourier component. In this process, we tune the parameters of cutting-off frequency and supertoroidal order to find the condition for space-time superoscillation.

For studying the spatial superoscillation, we construct a set of the spatiotemporal signal of band-limited supertoroidal-like pulses by equation (S26), for various values of cutting-off frequency f_m, supertoroidal order α, and extract the radial oscillating curve at t=0 and z=0. Then, we find all the zero points and calculate the distance between each pair of adjacent zero point, and the minimal value of which corresponds to the fastest radial half-cycle local oscillation, as shown in Figure S4. The fastest single-cycle radial local oscillation can be traced by similar way, i.e. the minimal distance across three adjacent zero points.

Similarly, for studying the temporal superoscillation, we construct a set of the spatiotemporal signal of band-limited supertoroidal-like pulses by equation (S26), for various values of cutting-off frequency f_m, supertoroidal order α, and extract the temporal oscillating curve at $r=10q_1$ and z=0. Then, we find all the zero points and calculate the distance between each pair of adjacent zero point, and the minimal value of which corresponds to the fastest half-cycle temporal local oscillation. The fastest single-cycle temporal local oscillation can be traced by similar way, i.e. the minimal distance across three adjacent zero points.

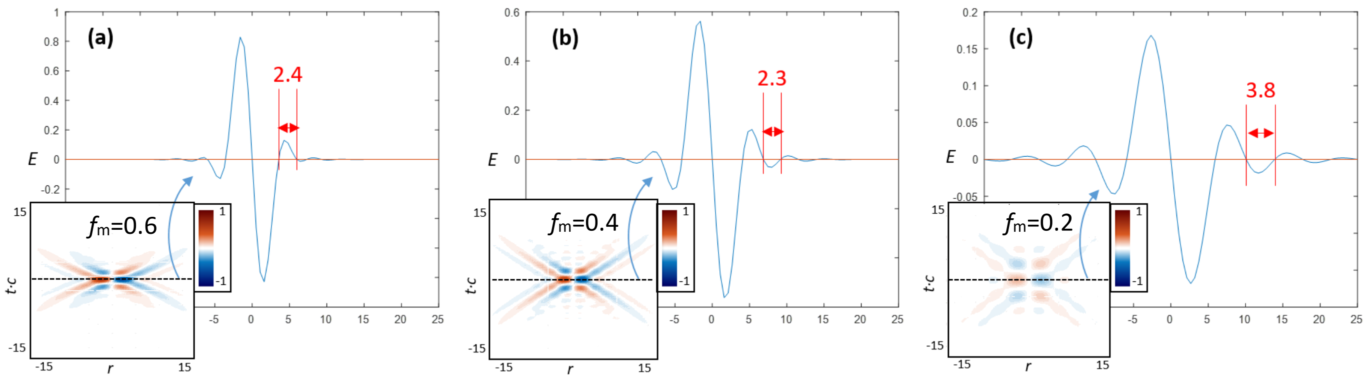

Figure S4: The examples of tracing the minimal radial oscillatory structures of band-limited supertoroidal-like pulses with cutting-off frequency set as (a) f_m = 0.6, (b) f_m = 0.4, (c) f_m = 0.2 (unit: c/q_1) at t=0 and z=0. The insets show corresponding space-time amplitude distributions. The results of minimal redial half-cycle oscillatory structure are marked in red with unit of q_1.

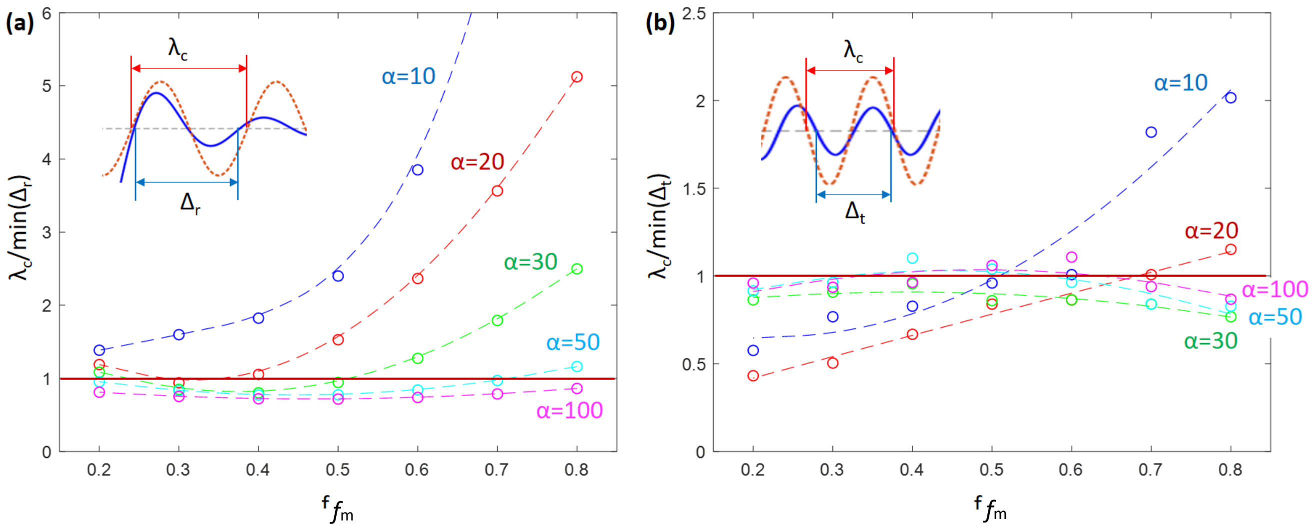

Figure S5: The results of (a) the ratio of cutting-off wavelength (of the fastest radial Fourier component) and the local minimal radial single-cycle oscillatory structure and (b) the ratio of cutting-off wavelength (of the fastest temporal Fourier component) and the local minimal temporal single-cycle oscillatory structure versus the cutting-off frequency f_m (f_m=0.2, 0.3, 0.4, 0.5, 0.6, 0.7, 0.8, unit: 1/q_1) for various values of supertoroidal order α (α=10, α=20, α=30, α=50, α=100). The red lines mark the threshold of superoscillation.

In order to qualitatively characterize the spatial superoscillation, we calculate the cutting-off wavelength (of the fastest radial Fourier component), $\lambda_m=c/f_m$, and the local minimal radial single-cycle oscillatory structure, noted as Δ_r, versus the cutting-off frequency f_m (f_m=0.2, 0.3, 0.4, 0.5, 0.6, 0.7, 0.8, unit: 1/q_1) for various values of supertoroidal order α (α=10, α=20, α=30, α=50, α=100), the results of which are shown in Figure S5(a). We can observe the evidence of spatial superoscillation that there are cases where λ_m/min(Δ_r) is less than 1. For a proper setting of cutting-off frequency, the higher value of supertoroidal order the easier to observe the spatial superoscillation (at the range we studied α<100). In order to qualitatively characterize the temporal superoscillation, we calculate the cutting-off wavelength (of the fastest temporal Fourier component), $\lambda_m=c/f_m$, and the local minimal temporal single-cycle oscillatory structure, noted as Δ_t, versus the cutting-off frequency f_m (f_m=0.2, 0.3, 0.4, 0.5, 0.6, 0.7, 0.8, unit: 1/q_1) for various values of supertoroidal order α (α=10, α=20, α=30, α=50, α=100), the results of which are shown in Figure S5(b). We can observe the evidence of spatial superoscillation that there are cases where λ_m/min(Δ_t) is less than 1. For a proper setting of cutting-off frequency, the ratio is firstly decreased, over the threshold of superoscillation, then increased versus the increasing of supertoroidal order. In these results, we can find the cases where spatial superoscillation and temporal superoscillation occur simultaneously. For instance, the cases that f_m=0.3, 0.4, 0.5 and α=20, f_m=0.2, 0.6, 0.7, 0.8 and α=50, and f_m=0.2, 0.7, 0.8 and α=100. These are evidences of space-time superoscillation,

References

[1] R. W. Ziolkowski, *Phys. Rev. A* **39**, 2005 (1989).

[2] R. W. Hellwarth and P. Nouchi, *Phys. Rev. A* **54**, 889 (1996).

[3] Y. Shen, Y. Hou, N. Papasimakis, and N. I. Zheludev, *Nat. Commun.* **12**, 5891 (2021).
